# Web-Based Interactive Tool to Identify Facilities at Risk of Receiving Patients with Multidrug-Resistant Organisms

**DOI:** 10.3201/eid2609.191691

**Published:** 2020-09

**Authors:** Rany Octaria, Allison Chan, Hannah Wolford, Rose Devasia, Troy D. Moon, Yuwei Zhu, Rachel B. Slayton, Marion A. Kainer

**Affiliations:** Vanderbilt University Graduate School, Nashville, Tennessee, USA (R. Octaria);; Healthcare Associated Infections and Antimicrobial Resistance Program, Tennessee Department of Health, Nashville (R. Octaria, A. Chan, R. Devasia, M.A. Kainer);; Centers for Disease Control and Prevention, Atlanta, Georgia, USA (H. Wolford, R.B. Slayton);; Vanderbilt University Medical Center, Nashville (T.D. Moon, Y. Zhu, M.A. Kainer)

**Keywords:** multidrug resistance, antimicrobial resistance, bacteria, MRSA and other staphylococci, healthcare-associated infections, patient transfers, computer software application

## Abstract

To identify facilities at risk of receiving patients colonized or infected with multidrug-resistant organisms (MDROs), we developed an interactive web-based interface for visualization of patient-sharing networks among healthcare facilities in Tennessee, USA. Using hospital discharge data and the Centers for Medicare and Medicaid Services’ claims and Minimum Data Set, we constructed networks among hospitals and skilled nursing facilities. Networks included direct and indirect transfers, which accounted for <365 days in the community outside of facility admissions. Authorized users can visualize a facility of interest and tailor visualizations by year, network dataset, length of time in the community, and minimum number of transfers. The interface visualizes the facility of interest with its connected facilities that receive or send patients, the number of interfacility transfers, and facilities at risk of receiving transfers from the facility of interest. This tool will help other health departments enhance their MDRO outbreak responses.

Antimicrobial resistance (AMR) is an urgent public health threat causing an estimated 2,868,700 infections and 35,900 deaths each year in the United States (*1*). Multidrug-resistant organisms (MDROs), including carbapenem-resistant *Enterobacteriaceae* (CRE), methicillin-resistant *Staphylococcus aureus* (MRSA), and organisms related to antimicrobial drug use and resistance, such as *Clostridioides difficile*, often are the causative agents in healthcare-associated infections (*1*,*2*). Studies show that these pathogens can colonize patients for extended periods of time (*3*). One study found that 38% of patients colonized with CRE were still colonized even a year after discharge from a facility (*4*); such patients can serve as reservoirs for MDROs in the community or in healthcare facilities.

Previous healthcare exposure is a known risk factor for MDRO infections (*5*,*6*). Older adults, patients with underlying medical conditions, and residents of long-term care facilities (LTCFs) are more likely to have multiple healthcare exposures, making them more likely to develop infections (*7*). Movement of patients across healthcare facilities can serve as a means of spreading MDROs across a community and introducing new pathogens into a region. Interfacility patient sharing has been associated with higher incidence of both CRE and *C. difficile* infections (*5*,*8*).

A mathematical modeling study found that facility-level infection prevention measures alone are insufficient to prevent transmissions (*9*). A coordinated approach to contain MDROs among interconnected healthcare facilities and public health reduced acquisition by 74% in a small network model over 5 years and 55% in a large network over 15 years (*9*). Beginning in 2017, the Centers for Disease Control and Prevention (CDC) issued guidance for state and local health departments and healthcare facilities to contain novel MDROs (*10*). The guidance classifies organisms into 3 tiers based on public health threat and outlines the recommended containment approach, which includes a coordinated approach among healthcare facilities, public health, and laboratories (*10*).

Despite numerous research publications on the role patient-sharing networks play in elucidating MDRO transmission, few address the application of these networks in public health practice. We used patient-sharing networks to design tailored strategies to help public health contain the spread of MDROs. We developed an interactive tool to visualize networks of patient sharing among hospitals and skilled nursing facilities (SNFs) in the state of Tennessee. Our tool enabled the Tennessee Department of Health (TDH) to identify facilities at risk of receiving patients suspected to be colonized with AMR pathogens.

## Methods

### Patient Matching

We constructed interfacility patient-sharing networks from the Tennessee Hospital Discharge Data System (HDDS) inpatient admissions, and Centers for Medicare and Medicaid Services (CMS) claims and Minimum Data Set (MDS; https://www.cms.gov). The HDDS dataset included all inpatient admissions to Tennessee acute-care hospitals (ACHs) licensed by TDH; admission to LTCFs and Department of Veterans Affairs hospitals were not captured in this dataset. We used HDDS data to summarize patient-sharing data among Tennessee facilities, including ACHs critical access hospitals (CAH), long-term acute-care hospitals (LTACH), and inpatient rehabilitation facilities (IRFs) from January 2014–December 2017. 

We linked admissions of each patient in the HDDS dataset with a multilevel matching process by using patient identifiers. First, we linked consecutive admissions for <365 days by matching the combination of date of birth, sex, and Social Security number (SSN). In this step, we considered admissions of the same person to be those that matched for date of birth, sex, and first and last name, even if the SSN was missing or had a 1-digit difference. Subsequently, we linked admissions that did not generate matches in the first step by matching the combination of date of birth, sex, and full name, even with >2-digit differences in the SSN. To protect patient privacy, patient-level admission data used for matching were saved in secured hard-drives that were connected to the computer only when generating facility-level data.

The CMS dataset included claims data and data from the MDS, which captured all inpatient admissions of CMS fee-for-service beneficiaries to Tennessee hospitals and SNFs. We used MDS admission and discharge assessments to identify all visits of Medicare beneficiaries to SNFs, a type of LTCF that is not as intensive as hospital but offers more intensive medical and nursing services, such as subacute care (*11*). We combined MDS visits with CMS claims data that included admissions in all types of hospitals in the HDDS to create a more complete dataset of visits for Medicare beneficiaries. We linked admissions in MDS to patients by matching the CMS beneficiary identification number. We aggregated facilities by using the facilities’ CMS certification number, which is different than facility aggregation in the HDDS dataset. The CDC modeling unit conducted aggregation by using the secure environment of the CMS Virtual Research Data Center before sharing the facility-level aggregate data to TDH.

### Network Construction

Because of differences in aggregation, we constructed the CMS and HDDS networks separately. The CMS dataset aggregated facilities based on their CMS certification number, but HDDS aggregated based on the assigned Tennessee state licensing registration. 

From each data source, we constructed 2 types of networks that connected healthcare facilities through uninterrupted patient sharing (UPS) and total patient sharing (TPS) (*12*). UPS, or direct transfers, connect a pair of facilities when a patient is discharged from 1 facility and admitted directly to another facility within 1 day. We accounted for patients who spent time in the community between healthcare admissions through the TPS network, which connects a pair of facilities through direct and indirect transfers. An indirect transfer occurs when a patient is discharged from 1 facility and readmitted to another facility within 2–365 days. The number of days between consecutive admissions was calculated by subtracting the next admission date and the current discharge date. We constructed subnetworks from the overall TPS network for 30 and 365 days in the community.

In our visualizations, each healthcare facility is represented by a node. A pair of nodes is connected by a line, also known as an edge in network analysis, weighted by the number of 1-way patient shares between pairs of facilities. For example, a patient discharged from a facility on September 30, 2015 and admitted to another on September 29, 2016, represents 1 indirect transfer. A patient can be represented by multiple edges in the same network. For example, if hospital A discharged Mr. X on January 30 and hospital B admitted Mr. X 2 weeks later, the TPS network graph would represent this connection as an edge with a weight of 1 going from node A to node B. If Mr. X is then admitted to SNF C 2 months later, this indirect transfer will be represented only as another edge from node B to C, but not A to C.

### Network Analysis

**Figure 1:**
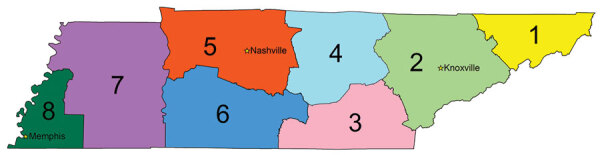
Emergency Medical Services (EMS) regions in Tennessee, USA: 1) Northeast; 2) East; 3) Southeast; 4) Upper Cumberland; 5) Mid-Cumberland; 6) South Central; 7) West; and 8) Memphis-Delta. The 8 EMS regions represent the referral patterns for EMS services and hospitals and for coordination for emergency preparedness activities. The Tennessee Department of Health uses EMS regions to aggregate multidrug-resistant organisms surveillance data. Stars indicate metropolitan areas within EMS regions.

We used an ego network design for the tool; this type of social network consists of a focal node (ego) and the nodes to which it is connected, directly or indirectly. In our tool, the facility of interest in each web session acts as the ego facility. We defined the following centrality measures for each ego facility by calendar year and by type of network: in-degree, out-degree, weighted in-degree, and weighted out-degree. We defined in-degree as the total number of facilities that sent transfers to a given facility and out-degree as the total number of facilities that received transfers from a given facility (*12*). We defined weighted in-degree as the total number of patient transfers sent to a given facility and weighted out-degree as the total number of patient transfers sent from a given facility (*8*). We used the Fruchterman-Reingold force-directed graph drawing algorithm to assign the relative positions of each facility in the network graph (*13*). We accounted for several characteristics of the healthcare facility, including the type of facility and the Emergency Medical Services (EMS) region in which the facility is located (Figure 1). Tennessee EMS regions represent the referral patterns of the EMS services and hospitals, and the coordinating areas for emergency preparedness activities, which TDH uses to aggregate MDRO surveillance data.

At-risk facilities targeted in public health containment efforts can vary based on the circumstances of each outbreak. For our purposes, we defined at-risk facilities as downstream facilities that historically were identified to have received patients from the ego facility. At-risk facilities also were classified as the facilities receiving the most historical transfers from the ego facility if there were >10 downstream facilities. To evaluate the long-term stability of these identified at-risk facilities in the HDDS network, we evaluated the top downstream facilities of 5 randomly selected ego facilities across different EMS regions from 2014–2017. For each ego facility, we compared the 5 downstream facilities receiving the most transfers between pairs of consecutive years to quantify the aggregate percent change in the top 5 downstream facilities.

### Web-Based Application

**Figure 2:**
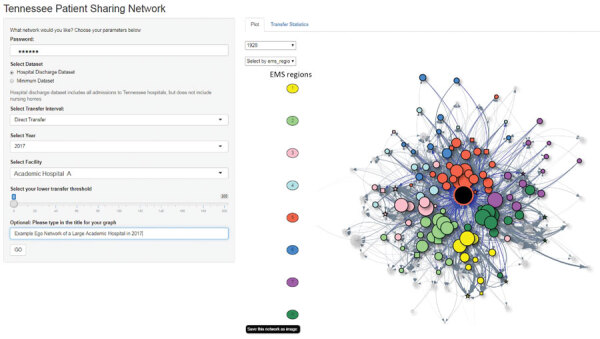
Screenshot of the initial user interface and network graph visualization tab of the web-based application developed to identify healthcare facilities at risk of receiving patients with multidrug-resistant organisms. The application was designed using Shiny (R Studio Inc., https://www.rstudio.com). The network graph is visualized by using a force-directed layout. Black node in the center indicates the facility of interest (ego facility). Tennessee EMS regions are represented by the node color for connected facilities and is represented by the color of the node border for the ego facility. Users can change visualizations interactively during real-time use. EMS, Emergency Medical Services.

We developed a password-protected web-based application using Shiny (R Studio Inc., https://www.rstudio.com) to enable public health personnel to access network visualizations and transfer statistics easily. Approved usernames and passwords are managed internally by TDH Healthcare Associated Infections and Antimicrobial Resistance (HAI/AR) program. Authorized users can access the Shiny web application to visualize the network of a facility of interest (ego) through a user-friendly interface at the website, https://tnhealthhai.shinyapps.io/patientsharing (Figure 2). The ego facility is the facility of interest that serves as the center of the visualized network for the current online session. Users can select from menus to tailor the displayed plot based on the data source, HDDS or MDS; year; length of interim time in the community; and the ego facility.

In the network plot, the node color represents Tennessee EMS regions, node size represents number of beds, and node shape represents facility type. The thickness of the edge is weighted on the number of 1-way transfers, including multiple transfers of 1 patient, between a facility pair. When users place the cursor over a node, the tooltip function displays the facility name, facility type, and number of beds. A slider widget enables users to set the lower threshold of 1-way transfers between each pair of facilities displayed for the session (Figure 2).

**Figure 3:**
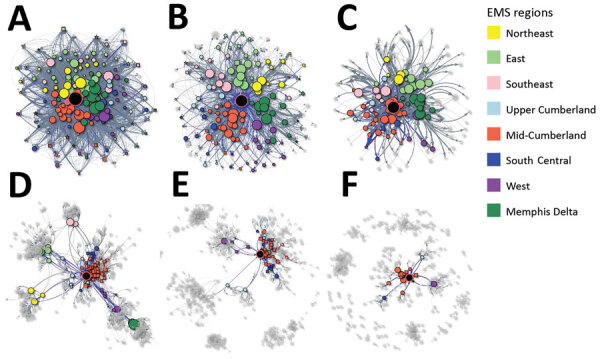
Varying user-tailored ego network visualizations in the web-based interactive tool to identify facilities at risk of receiving patients with multidrug-resistant organisms. Panels demonstrate options for visualizations for a large academic hospital from the HDDS and Centers for Medicare and Medicaid claims and MDS. Real-time use of the application enables users to tailor visualizations by facility, patient transfer threshold, and type of network. Black node in the center indicates the facility of interest (ego facility). The EMS region is represented by the node color for connected facilities and is represented by the color of the node border for the ego facility. Displays shown use the HDDS dataset (A–C) and MDS dataset (D–F). Panels A and D demonstrate a total patient sharing network; B and E demonstrate an uninterrupted patient sharing network; C and F are examples of alterations in patient threshold transfers and displays facilities that have >50 patient transfers to or with the ego facility. EMS, Emergency Medical Services; MDS, minimum dataset; HDDS, Tennessee Hospital Discharge Data System.

The Shiny application has 2 display tabs, plot and transfer statistics. The plot tab displays a visualization of the ego-network and all facilities that shared patients with the ego facility (Figure 3). When users hover the cursor over an edge, the application displays the number of 1-way transfers. Users can interact by applying filters for region or facility of interest, and by dragging the position of different nodes.

**Figure 4:**
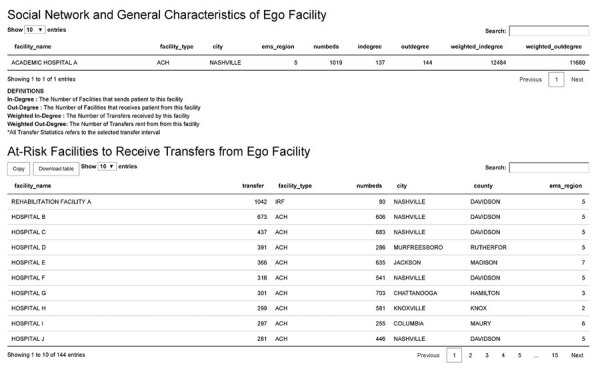
Screenshot of the transfer statistics tab of the web-based interactive tool to identify facilities at risk of receiving patients with multidrug-resistant organisms. This function displays facility characteristics and downstream facilities that are most likely to receive transfers from the ego facility. The second tab of the application’s user interface includes 2 tables. The top table displays detailed social network and facility characteristics for the ego facility. The bottom table displays the facilities at highest risk to receive patients from the ego facility, which are downstream facilities. The table defaults to sort the number of patient transfers in descending order. Users can interactively choose a column to sort and filter this table, which can be used to identify facilities at risk during outbreaks or regional detection of a novel organism. Hospital names have been de-identified for privacy.

The transfer statistics tab displays facility-level characteristics and facilities most at risk to receive transfers from the ego facility (Figure 4). It also lists the ego facility’s type, city, EMS region, number of licensed beds, and centrality measures and displays a table of facilities at risk to receive transfers from the ego facility. The list defaults to a descending order of facilities by the number transfers from the ego facility. Users can filter or sort the table display based on facility name, facility type, number of beds, county, and EMS region. A download button allows users to import the table as comma-separated values, or as Microsoft Excel (https://www.microsoft.com) or portable document format (PDF) files.

### Software

Data cleaning and person-matching were completed in SAS 9.4 (SAS Institute, Cary, NC, USA). We conducted network analyses by using the Statnet and network visualization by using visNetwork packages, both in Rstudio version 3.5.2 (R Studio Inc.) (*14*,*15*). As described previously, we developed the interactive web-based network visualization application by using Shiny. We uploaded de-identified facility-level datasets to the shinyapps.io server hosted on Amazon Web Services (Amazon, https://aws.amazon.com) infrastructure in the United States. These datasets had facility-level patient transfer statistics and characteristics, including licensed facility names, number of beds, facility type, and city and county of address.

### Ethics Considerations

The patient-sharing network project was exempted from the institutional review boards (IRBs) at CDC (IRB no. 032416JO), TDH (IRB no. 923990-1), and Vanderbilt University (IRB no. 161676). This work was conducted under a data use agreement between CDC and CMS. CDC’s Human Research Protection Office determined this project was exempt from regulations governing the protection of human subjects in research under 45 CFR 46.101(b).

## Results

The Shiny web application includes facility-level patient sharing data from the 2014–2017 HDDS dataset and Medicare datasets from 2014 and 2016. Both data sources had 3 networks for each transfer interval: direct, 30, and 365 days. The 2017 HDDS network included a total of 146 hospitals of 4 types; 116 ACH, 13 CAH, 9 IRF, and 8 LTACH. In 2017, the HDDS dataset recorded 886,277 inpatient hospitalizations representing 494,153 patients. Among patients discharged from Tennessee hospitals in 2017, a total of 29.5% (145,953) were readmitted to another Tennessee hospital within 365 days. The median interval of time in the community was 46 (IQR 11–109) days; 13.8% of patients who were readmitted to a different hospital were directly transferred.

The 2016 CMS dataset reported 381,627 stays to 465 Tennessee facilities representing 196,528 unique patients. These 465 facilities included 91 ACHs, 16 CAH, 10 LTACHs, 10 IRFs, 322 SNFs, 1 children’s hospital, and 15 psychiatric hospitals, as classified by the CMS certification numbers. Among all admissions to a Tennessee facility, 82.9% (316,368) of patients had a previous healthcare admission within 365 days; 29.9% of those readmissions were a direct transfer from a healthcare facility. The median interval of time in the community was 11 (IQR 0–108) days. Our downstream facility analysis showed that among the 5 randomly selected facilities, 84% (range 80%–88%) of the facilities in the top 5 from the prior year were in the top 5 again in the succeeding year (Appendix). 

## Discussion

TDH has used this interactive tool to improve statewide awareness of the importance of interfacility connectedness, particularly during outbreaks and for containment responses of novel MDROs. We designed the tool as a web-based application for real-time, easy access with internet browsers from computer desktops or handheld devices. This flexibility ensures public health staff can access the application to identify at-risk facilities in a variety of settings, such as when in the field performing point prevalence surveys or during routine office work. The application has helped epidemiologists and infection preventionists prioritize communication during public health containment responses.

We have demonstrated that a facility’s ego network can accurately predict the facilities patients visit after discharge from the index facility during an outbreak (*6*). The TDH HAI/AR team used the application during a particular multifacility outbreak that had evidence of MDRO interfacility and intrafacility transmission. The tool allowed us to identify facilities that frequently received patients from the 2 ego facilities involved in the outbreak. TDH alerted these downstream facilities, which led them to consider admission screening for incoming patients from the 2 ego facilities. TDH plans to continue to use this tool during similar outbreaks. Facility transmission warrants public health action to alert downstream facilities to consider admission screening or enhanced contact precautions for patients admitted from the ego facility.

In addition, the TDH HAI/AR team introduced and demonstrated the use of this application to infection preventionists at hospitals and nursing homes through a variety of webinars and in-person presentations across the state. TDH received requests from facility infection preventionists for line lists of downstream facilities because they were planning containment efforts and wanted to understand which facilities receive the most patients from their facilities. TDH did not provide hospital infection preventionists access to the application but fulfilled requests by emailing exported line lists as Excel documents. Information on downstream facilities can help inform which facilities to target for relationship development and likely will assist with communication during patient transfers. 

The TDH HAI/AR team performs targeted infection control assessments as part of a MDRO prevention strategy. These assessments, conducted by TDH infection preventionists, are nonregulatory, consultative, on-site healthcare facility visits to identify gaps in infection prevention specific to a targeted pathogen or area of concern. Our web-based Shiny application was and will continue to be used to identify highly connected facilities in each EMS region and across the state. Prior studies found a correlation between the incidence of MDROs in a healthcare facility and the facility level of connectedness, measured by weighted in-degree and out-degree (*6*,*8*). Identification of highly connected facilities is valuable because it enables us to perform preemptive targeted infection control assessments before the introduction and spread of MDROs. Public health staff can assist by ensuring adequate infection prevention practices are in place at highly connected facilities where a potential for catalyzing interfacility transmission exists.

Our patient-sharing network has several strengths. Access to the hospital discharge data and granular patient identifiers enabled us to conduct person matching with high-level identifiers. We were able to use a robust method to match patients from populations with any insurance coverage for statewide data in HDDS. Moreover, the use of 2 complementary datasets established a highly inclusive picture of a facility’s ego network. With both the HDDS and CMS datasets, ACHs, CAHs, IRFs, LTACHs, and SNFs could be included in our analyses each time an ego facility is evaluated. Previously published patient-sharing network analyses were constructed by using partial data that included only direct patient transfers; a subset of patient population, such as CMS beneficiaries; hospitals; or county-level data (*6*,*8*,*12*,*16*).

The inclusion of SNFs was critical for analysis because LTCFs are a key component to a hospital’s patient sharing network (*17*). Although not all types of LTCFs were included our network, the inclusion of SNFs represent the facilities carrying a considerable burden of MDRO infections. Point prevalence analysis of MDS data found that MDRO infections were found in 4.2% of nursing home residents in the United States (*18*). Colonization with MDROs were found to be more common among nursing home residents (*19*,*20*). Smaller cohort studies showed gram-negative bacteria was found in 39% of nursing home residents and MRSA was found in 42% (*19*,*21*). Thus, communication with LTCFs is crucial for outbreak management and prevention activities.

 An additional strength of the application is its built-in flexibility, which allows the user to tailor the colonization period for specific organisms. The inclusion of 365 days as the longest transfer period for indirect transfers reflects the documented colonization period of CRE in the community (*22*), but users can change this parameter to account for MDROs with shorter colonization periods. The application also can display facilities connected only through direct transfers or through 30-day indirect transfers, which might reflect the colonization period of different MDROs more closely.

One limitation is the construction of 2 separate networks with the HDDS and CMS datasets. Ideally, the tool would include 1 large network with all facilities, but the construction of separate HDDS and CMS networks was required because of the differences in facility aggregation. Although both networks include ACHs, the unique number varies in each because of the difference in aggregation. Aggregating facility-level transfer data together might result in loss of information about some granular patient-sharing patterns in HDDS. One CMS certification number from the datasets can represent a group of tertiary hospitals within the same organization, creating a challenge to merge these data with the HDDS database. More recent CMS datasets include ZIP code information and the CMS certification number. We hope to use this additional datapoint in future analyses while exploring facility aggregation and standardization strategies for our databases.

We will continue to develop and improve the application with the addition of upstream facilities. We will update the network data and facility characteristics for the application annually with the most recent HDDS and CMS data. We also plan to develop models to outline the risk for transmissions based on their relative position in the network. We are working to merge the HDDS and CMS network data by standardizing facility identifications for a unified patient sharing network that provides a more complete picture of the patient population. Finally, we plan to expand the availability of this web-based platform to other public health departments by developing a feature to allow for external data uploads so health department staff can visualize their regional patient transfer networks. Access to information on patient-sharing networks would assist public health departments in mitigating MDRO transmission in their jurisdictions.

Appendix Deidentified names of Tennessee healthcare facilities included in downstream facility analysis to evaluate stability of patient sharing network across years.
